# Mitochondria-Associated Endoplasmic Reticulum Membranes: Inextricably Linked with Autophagy Process

**DOI:** 10.1155/2022/7086807

**Published:** 2022-08-23

**Authors:** Chonghao Ji, Zhanwei Zhang, Zechuan Li, Xiao She, Xiaoya Wang, Binyang Li, Xin Xu, Dawei Song, Dongjiao Zhang

**Affiliations:** ^1^Department of Implantology, School and Hospital of Stomatology, Cheeloo College of Medicine, Shandong University, Jinan, China; ^2^Shandong Key Laboratory of Oral Tissue Regeneration, Jinan, China; ^3^Shandong Engineering Laboratory for Dental Materials and Oral Tissue Regeneration, Jinan, China; ^4^School of Stomatology, Shandong First Medical University & Shandong Academy of Medical Sciences, Tai'an, China

## Abstract

Mitochondria-associated membranes (MAMs), physical connection sites between the endoplasmic reticulum (ER) and the outer mitochondrial membrane (OMM), are involved in numerous cellular processes, such as calcium ion transport, lipid metabolism, autophagy, ER stress, mitochondria morphology, and apoptosis. Autophagy is a highly conserved intracellular process in which cellular contents are delivered by double-membrane vesicles, called autophagosomes, to the lysosomes for destruction and recycling. Autophagy, typically triggered by stress, eliminates damaged or redundant protein molecules and organelles to maintain regular cellular activity. Dysfunction of MAMs or autophagy is intimately associated with various diseases, including aging, cardiovascular, infections, cancer, multiple toxic agents, and some genetic disorders. Increasing evidence has shown that MAMs play a significant role in autophagy development and maturation. In our study, we concentrated on two opposing functions of MAMs in autophagy: facilitating the formation of autophagosomes and inhibiting autophagy. We recognized the link between MAMs and autophagy in the occurrence and progression of the diseases and therefore collated and summarized the existing intrinsic molecular mechanisms. Furthermore, we draw attention to several crucial data and open issues in the area that may be helpful for further study.

## 1. Introduction

Mitochondria and endoplasmic reticulum (ER) are two organelles with incredibly intricate structures and functions in eukaryotic cells. The mitochondrion supplies energy for physiological activities [1, 2], and ER is involved in the synthesis, modification, transport, secretion of protein macromolecules, and modulation of Ca^2+^ signaling [3, 4]. Bernhard et al. discovered a physical connection between the ER and mitochondria in the 1950s for the first time on electron micrographs of rat liver cells in 1952 and 1956 [5–7]. Restricted by biological technology development, it was not until 1990 that Jean Vance isolated and biochemical described the mitochondria-associated membranes (MAMs) from rat liver, which were initially called “fraction X” [8].

Increasing evidence has shown that these connections exist in various eukaryotic cells, including neural, reproductive, and mesenchymal stem cells with their composition and function being investigated in depth. As a platform for interorganelle communication between the subdomain of the ER and the outer mitochondrial membrane (OMM), the MAMs are dynamically connected by a series of molecules. These structures perform various functions, mainly relying on proteins engaged in cellular processes, such as Ca^2+^ transport, lipid metabolism, inflammasome formation, autophagy, ER stress, and mitochondria morphology, [9–14] of which the relationship between MAMs and autophagy is the focus of this review.

Microautophagy, chaperone-mediated autophagy (CMA), and macroautophagy, the latter of which is more commonly known as “autophagy,” are the three primary subcategories of this evolutionarily conserved process [15, 16]. In macroautophagy, cellular contents are transported by autophagosomes, performed as double-membrane vesicles, to lysosomes for destruction and recycling, thereby promoting normal cellular life activities [17–19]. According to the available research, yeast's autophagic mechanism differs from that of mammalian cells while yet being comparable to it. However, the molecular mechanisms underlying the initiation and expansion of autophagosomes have not been fully elucidated. Previous studies have found that MAMs are closely related to autophagy development and maturation. Here, we presented the evidence supporting that MAMs play essential roles in autophagy. It is important to note that in this review, autophagy generally refers to macroautophagy in mammalian cells, with alternative mechanisms being expressly mentioned as necessary.

## 2. Protein Composition of MAMs

Proteins in MAM fragments have recently been identified using a variety of techniques, including mass spectrometric analysis and peroxidase-mediated proximity biotinylation, and it was discovered that about 100 proteins were localized to the MAMs [11, 20, 21].

**Figure 1:**
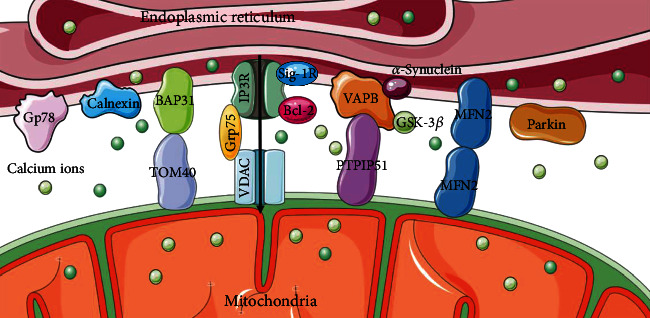
The primary molecular composition of MAMs. IP3R-Grp75-VDAC, VAPB-PTPIP51, and BAP31-TOM40 are protein complexes, and Sig-1R, Bcl2, MFN2, Gp78, calnexin, and Parkin are individual proteins involved in MAMs that form the influential contacts with each other.

IP_3_R-Grp75-VDAC is one of the most important groups of proteins involved in ER-mitochondria coupling [22]. The inositol 1,4,5-triphosphate receptor (IP_3_R) in the endoplasmic reticulum membrane and voltage-dependent anion channel (VDAC) in the OMM are Ca^2+^-related proteins that mediate the transport of Ca^2+^, thus affecting cellular metabolism and autophagy [23, 24]. As a molecular chaperone, glucose-regulated protein 75 kDa (Grp75) binds to IP_3_R and VDAC (Figure 1), maintaining the structural stability of the interaction and ensuring stable Ca^2+^ transport [25].

Moreover, the VAPB-PTPIP51 interaction is another group of proteins located on MAMs [26]. A C-terminal transmembrane domain anchors vesicle-associated membrane-associated protein B (VAPB) to the ER membrane. Protein tyrosine phosphatase-interacting protein 51 (PTPIP51), found in the OMM, can attach to the free N-terminus of VAPB in the cytoplasm [27, 28]. They form protein complexes to mediate calcium ion transport and autophagy, which can be regulated by other proteins, such as *α*-Synuclein and glycogen synthase kinase-3*β* (GSK-3*β*). Mutations in *α*-synuclein disrupt the VAPB-PTPIP51 complex, leading to the uncoupling of MAMs and subsequently resulting in dysregulation of Ca^2+^ transport [29]. Activation of GSK-3*β* diminishes the association of the VAPB-PTPIP51 complex, causing a disturbance in the dynamic equilibrium of Ca^2+^ ions [30]. Accumulation evidence suggests that overexpression of VAPB or PTPIP51 increases MAMs and reduces autophagosome formation [31, 32].

Located at the ER membrane, B cell receptor-associated protein 31 (BAP31) is a transmembrane protein that participates in the endoplasmic reticulum-associated degradation (ERAD) pathway and apoptosis [33], which can interact with a variety of proteins on the OMM to conduct a variety of functions. The outer mitochondrial membrane 40 (TOM40) in OMM is the translocation enzyme complex that promotes external protein translocation to mitochondria [34]. The interaction between BAP31 and TOM40 facilitates the pre-NDUFS4 transfer from the cytoplasm to the mitochondria, which enhances the nuclear-encoded mitochondrial protein translocation and oxygen consumption [35]. Moreover, the association of BAP31 and CDIP1, a proapoptotic p53 target, is improved upon ER-stress-mediated apoptosis. Bax oligomerization is brought on by the activation of caspase-8, CDIP1-dependent truncated Bid (tBid), and the recruitment of Bcl2 to the BAP31-CDIP1 complex. As a result, the Bax-dependent mitochondrial apoptotic pathway is enhanced [36]. The mitochondrial fission protein Fission 1 homologue (Fis1) communicates with BAP31 to convey apoptosis signals from the mitochondria to the ER. Apoptosis is facilitated by Fis1-promoted cleavage of BAP31 to proapoptotic p20BAP31 and procaspase-8 recruitment [37].

The molecular composition of MAM-associated lipid microdomains and their role in regulating and impacting numerous cellular functions have been revealed by a rising number of proteomic studies [38]. Indeed, several MAM proteins have been reported to be localized in lipid rafts, including the sigma-1 receptor (Sig-1R), progerin 2 (PSEN2), and reticulon 1 (RTN1) [39, 40]. Consequently, its regulation is closely associated with lipid transportation, specifically cholesterol delivery. The proteins Sig-1R and the B-cell lymphoma-2 (Bcl2) family, which have also been discovered to be enriched in MAMs, influence the Ca^2+^ transport between the ER and mitochondria [41, 42].

A key player in the coupling of the ER and mitochondria, the GTPase Mitofusin2 (MFN2) also regulates respiration, autophagy, and mitochondrial movement [43–45]. Under MFN2-knockout conditions, separation of ER and mitochondria was observed in mouse embryonic fibroblasts, neurons, and cardiomyocytes by TEM images [46, 47]. However, it was doubted that the siRNA knockdown of MFN2 facilitated the coupling of ER and mitochondria [48]. Furthermore, differential mediation of MFN2 to MAMs depends on mitochondrial location and type of communication [49]. Parkin is a protein whose overexpression maintains ER-mitochondria tethering and Ca^2+^ homeostasis. The absence of Parkin decreased ubiquitylation of MFN2 altering MAMs' integrity [50, 51], demonstrating that Parkin is associated with mitophagy [52].

Gp78 is a membrane-bound glycoprotein with a molecular weight of 78 kDa and an E3 ubiquitin ligase anchored in the ER [53] and has been confirmed to be located in MAMs and interacted with PINK1 involving mitophagy [54, 55]. Calnexin (CNX) is involved in protein folding and is required for autophagy, selectively recognizing misfolded endogenous procollagens in the ER [56, 57]. The role of these proteins implies an association of MAM with autophagy.

More and more proteins have been declared in exploring the structure of MAMs. It is important to note that they interact and couple with one another rather than being in isolation, maintaining the variety of the biological processes and composition of MAMs.

## 3. MAMs and Autophagy

Autophagy is a dynamic process composed of five steps, including autophagosome initiation, elongation, maturation, autophagosome-lysosome fusion, and the degradation of intra-auto-phagosomal contents by lysosomal hydrolases [58]. Specifically, the Atg1/ULK1-containing complex (containing Atg1, Atg13, and Atg17) participates in forming an omegasome after the induction of autophagy. Following that, the class III PI3K-Atg14 complex, the Atg9-Atg2-WIPI-1 (Atg18) complex, and the Atg5-Atg12-Atg16L1 complex regulate the formation of an isolation membrane called phagophore, which elongates to enclose and form an autophagosome in the presence of lipidated microtubule-associated protein 1 light chain 3 (MAP1LC3, also known as LC3). The lipidation of LC3/GABARAP depends on ATG3, ATG4, and ATG7. These autophagosomes are transported and fuse with the lysosome to form an autolysosome containing hydrolases to degrade intra-auto-phagosomal cargo [59–63], and these stages of autophagy are regulated by a series of molecules, including proteins in MAMs.

### 3.1. MAMs Are Involved in the Origin of Autophagosomes

**Figure 2:**
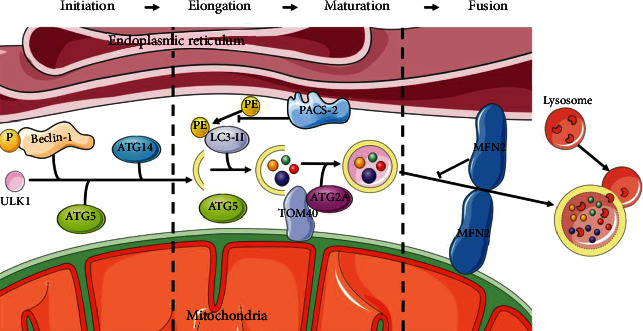
MAMs provide platforms to develop autophagosomes. The initiator of autophagosome ULK1 interacts with phosphorylated Beclin-1, ATG14, and ATG5 that accumulate in MAMs to form the phagophore. ATG2A involves the enlargement of autophagosomes depending on TOM40. PACS-2 inhibits the lipidation of LC3. MFN2 is the blocker of autolysosome formation.

Partially membranous organelles are generally accepted as the primary source of autophagosomal membranes. In particular, the current studies suggest that autophagy begins at the ER-mitochondria coupling site (Figure 2). In the initiation of phagophore expansion, starvation triggered the translocation of unc-51-like autophagy activating kinase 1 (ULK1) to the MAMs, thereby activating phosphorylation of the downstream autophagy effector protein Beclin-1 [64, 65]. Also, in this step of autophagy, the content of ATG14, a component of the phosphatidylinositide 3-kinase (PI3K) complex, progressively relocalizes to the MAMs under starvation conditions. At the same time, ATG5, an autophagosome-formation marker, is transferred to the MAMs until the formation is complete [66]. During elongation, ATG2A, a subtype of ATG2, translocates from the MAMs to the phagophore to enlarge the autophagosome and enhance autophagic flux, dependent on the mitochondrial translocase of outer membrane (TOM) components TOM40 and TOM70 [67, 68]. Phosphofurin acidic cluster sorting 2 protein (PACS-2) is a protein involved in MAMs integrity whose absence inhibits the lipidation of LC3/GABARAP, resulting in the inhibition of autophagy [69, 70]. The depletion of MFN2, one of the major modulators of MAMs, has been shown to impair membrane formation or block fusion in the autophagy process [71].

### 3.2. MAMs Positively Regulate Autophagy

**Figure 3:**
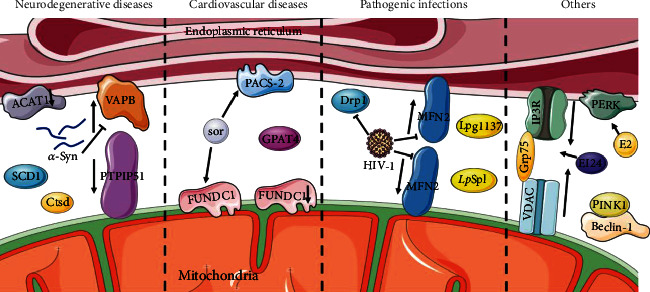
MAMs are positively correlated with autophagy. In neurodegenerative diseases, the blockage of ACAT1 elevates the local cholesterol levels in the MAMs to promote autophagosome formation. SCD1 and Ctsd are enriched in MAMs and promote autophagy. Pathogenic mutation of *α*-Syn decreases the association of the VAPB-PTPIP51 complex and disrupts autophagy. Reduction of FUNDC1 or activation of GPAT4 in MAMs inhibits autophagy and causes cardiovascular disease. The sor increases PACS2 and FUNDC1, activates autophagy, and causes cardiac dysfunction. HIV-1 impairs MAMs and blocks autophagic flux by reducing MFN2 and Drp1. Legionella pneumophila secretions Lpg1137 and LpSpl target MAMs to regulate autophagy. Interactions of PINK with Beclin-1, EI24 with IP3R-Grp75-VDAC complex, and E2 with PERK promote autophagy.

Positive regulation of autophagy by MAMs has been observed in various diseases (Figure 3).

Cell death in the nervous system may be triggered in neurodegenerative diseases by impaired autophagy brought on by a malfunction in the MAMs or any tethering protein, according to ongoing research. Cholesterol acyltransferase 1 (ACAT1), utilized as a marker for MAMs, is abundant in MAMs [39]. Some researchers speculated that the blockage of ACAT1 might elevate the local cholesterol levels in the MAMs to promote autophagosome formation [72]. Then, in Alzheimer's disease (AD) neurons, the activated autophagy eventually accelerates the degradation of A*β*42 [73], implying that MAMs are crucial to the biogenesis of autophagosomes.

*PARK2* and *PARK6* encode Parkin and phosphatase and tensin homolog (PTEN)-induced kinase 1 (PINK1), whose mutations were reported in Parkinson's disease (PD) [74, 75]. Parkin is an E3 ubiquitin ligase that contributes significantly to mitophagy and maintains the integrity of MAMs [76]. Resveratrol treatment of Parkin-mutant fibroblasts showed a higher expression of proteins implicated in the tethering of ER and mitochondrial contact sites and caused an enhanced autophagic flux. The mechanisms were hypothesized that resveratrol modulated the pAMPK/AMPK pathway to stimulate the Ca^2+^ level [77]. The pathogenic mutation in *α*-Syn, one kind of PD-related gene, leads to pathological changes in MAMs, decreases its association, and disrupts autophagy [78–81]. In addition, PINK1-induced alterations in MAMs have been observed in renal ischemia-reperfusion (I/R) [82]. It has been demonstrated that the contact between PINK1 and the preautophagic protein Beclin1 could increase MAMs and regulate mitophagy, thereby helping relieve renal I/R injury by regulating mitophagy [83, 84]. PINK1 was reported to phosphorylate MFN2 to facilitate the binding of MFN2 and Parkin for neonatal autophagosome recognition [85].

Moreover, reduced autophagy levels and MAM dysfunction were observed in human aging or senescence processes [86]. Stearoyl-CoA desaturase 1 (SCD1), a critical lipid metabolism enzyme enriched in MAMs, colocalizes with DGAT2, whose levels have been shown to decrease in the skin of aged people. In accordance with this, the inhibition of SCD1 impaired autophagosome biogenesis and affected autophagosome-lysosome fusion [87]. ZiBuPiYin recipe (ZBPYR) is a Chinese herbal remedy that counteracts the psychological stress- (PS-) induced diabetes-associated cognitive decline (PSD) [88, 89]. One of the brain MAMs proteins in PSD rats, Cathepsin D (Ctsd), is an autophagy-related protein downregulated in the ZBPYR treatment [89].

Numerous investigations have established that aberrant MAM levels, composition, or activity has a role in the pathophysiology of cardiovascular disease (CVD), perhaps through their modulation of autophagy. FUN14 domain containing 1 (FUNDC1), enriched at MAMs, acts as a mitophagy receptor, recruiting autophagosomes, and thus degrades damaged or excessive mitochondria [90, 91]. Patients diagnosed with heart failure (HF) exhibit a reduction of FUNDC1 [92]. The formation of MAMs is increased in cardiomyocytes of a type 2 diabetes mellitus mouse model suffering from diabetic cardiomyopathy [93]. In vascular smooth muscle cells (VSMCs), saturated lysophosphatidic acids (LPAs) are accumulated through the activation of glycerol-3-phosphate acyltransferase 4 (GPAT4) at the contact location between omegasomes and the MAMs. And this accumulation inhibited autophagic flux, leading to the development of severe vascular calcification [94]. The oncological therapeutical drug sorafenib (sor) reduced the MFN2 level in cardiomyocytes, elevated MAMs formation, increased mitochondria-MAM tethering, and activated autophagy. The sor treatment led to cardiac dysfunction and autophagy activation in the vivo model [95].

MAMs and autophagy may be targeted in pathogenic infections to undermine host cellular defense mechanisms, according to an underappreciated body of research. In response to HIV-1, MFN2 and Drp1 decreased with the impairment of ER-mitochondrial interaction, and a blockade of autophagy flux was observed [96, 97]. *Legionella pneumophila* secreted a serine protease Lpg1137, localized to the cytosol and the MAMs to degrade syntaxin 17 (STX17) in fed cells [98]. STX17 was identified as a promoter of the formation of autophagic vesicles. STX17 interacts with ATG14 before recruiting the PI3K complex to the MAMs in autophagosome biogenesis [66]. *Legionella* effector sphingosine-1 phosphate lyase activity (*Lp*Spl) is a T4SS secreted effector from *L. pneumophila* that appears to target MAMs to modulate the autophagy response to infection [99, 100].

Recently, other diseases have also explored the intrinsic mechanisms associated with MAMs. In breast cancer cells, 17*β*-estradiol (E2) therapy initiates the unfolded protein response (UPR), thereby activating autophagy and PRK-like endoplasmic reticulum kinase (PERK) in cancer cells, which is enriched at MAMs [101, 102]. In pancreatic *β* cells, an essential autophagy protein, etoposide-induced protein 2.4 (EI24), is observed abundantly in the MAMs to interact with IP_3_R-Grp75-VDAC and is necessary for the integrity of the MAMs and autophagy flux [103, 104].

Oxidative phosphorylation (OxPHOS) is one of the critical catabolic pathways for proliferation and growth in acute myeloid leukemia (AML) cells [105]. Autophagy degrades LDs supplying free fatty acids (FFAs) to mitochondria for fueling OxPHOS. It is interesting to note that MAM production is regulated by the activity of the mitochondrial respiratory chain, which in turn controls autophagy. And disruption of MAMs decreases autophagy, leading to lipid droplet (LD) accumulation in leukemia [106].

The ganglioside GD3 and ER lipid raft-associated protein 1 (ERLIN1) are lipid raft proteins in MAMs that have been detected in a molecular interaction with autophagy and beclin 1 regulator 1 (AMBRA1). The ST8SIA1 gene for ganglioside formation synthase was knocked down, which hampered autophagy and reduced GD3 or ERLIN1 activity at the MAMs level [107–109]. In patient-derived fibroblasts with remethylation disorders causing homocystinuria, overexpressed MAM-associated proteins and activation of the autophagy process were found [110].

### 3.3. MAMs and the Negative Regulation of Autophagy

**Figure 4:**
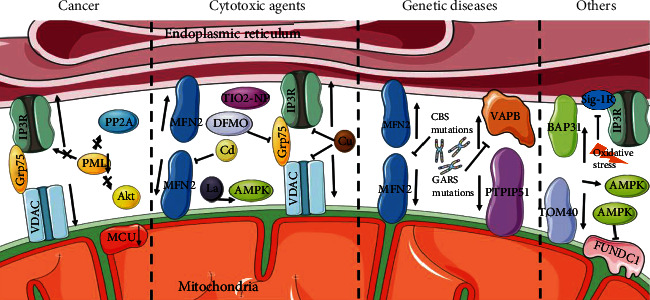
Negative correlations between MAMs and autophagy. In cancer cells, MAM inhibition or MCU reduces active autophagy. PML coordinates a complex with IP3R3, Akt kinase, and phosphatase PP2A. The loss of PML of MAMs results in autophagy activation. Cd, La, Cu, and Ti reduce different tethering protein complexes' expression of MAMs but enhance the autophagic protein levels. DFMO treatment reduces the expression of Grp75 and enhances autophagic flux. The mutations of GARS or CBS both downregulate MAM-related proteins but enhance autophagy. BAP31-TOM40 disruption stimulates autophagy by activating AMPK. AMPK reduces the expression of FUNDC1 and initiates autophagy. Oxidative stress conditions inhibited Sig-1R to promote autophagy.

Conversely, several other studies have reached contrasting conclusions regarding the relationship between MAMs' integrity and the extent of autophagy (Figure 4).

Cancer cells invariably exhibit an increase in autophagic flux upon IP3R inhibition or inducing autophagy by reducing the mitochondrial Ca^2+^ uniporter (MCU) [111]. A tumor suppressor promyelocytic leukemia protein (PML) was found to coordinate a complex with IP_3_R3, Akt kinase, and phosphatase PP2A in MAMs [112]. The loss of PML of MAMs resulted in autophagy induction. By reducing AMP-activated protein kinase (AMPK) activity in PML-KO cells, overexpressing MCU increased mitochondria's capacity to accumulate Ca^2+^ and suppressed autophagic flux [113, 114]. The interruption of ER-mitochondrial Ca^2+^ transfer in cancer cells could cause localized autophagy activation by AMPK present at the MAMs, followed by increasing Beclin-1 expression [115].

Metallic ions including cadmium (Cd), lanthanum (La), copper (Cu), and titanium (Ti) are all associated with a decrease in the expression of MAMs' tethering protein complexes, a reduction in their connections or colocalization, but an increase in the levels of autophagic proteins. Mfn2 defects in PC12 cells or primary neurons block the colocalization of ER and mitochondria while decreasing the Cd-induced enhancement of autophagic protein levels [116]. Lanthanum has neurotoxicity, which is expected to impair learning and memory ability. In offspring rats, LaCl_3_ decreased tethering protein complexes expression of MAMs, damaged MAMs structure, and upregulated NOX4 expression leading to an active ROS-AMPK-mTOR signaling pathway which induced autophagy [117]. Excessive Cu decreased the colocalization of IP_3_R and VDAC1, causing MAMs dysfunction and evoking autophagy in duck renal tubular epithelial cells [118]. TiO_2_-NP is an unidentified toxic nanomaterial that increases autophagy by upsetting the equilibrium of calcium ions and MAMs in human bronchial epithelial cells [119].

Thapsigargin is a sarcoplasmic/endoplasmic reticulum Ca^2+^ ATPase (SERCA) inhibitor that induces cell death in HAP1 cells [120]. SEC24A was identified as an adaptor protein during COPII-mediated vesicle transport and an essential novel mediator of thapsigargin-induced cell death [121]. Thapsigargin-treated SEC24A-inhibition cells showed fewer contacts between the ER and mitochondria, impairing Ca^2+^ efflux from ER and influx into mitochondria, but increasing autophagic flux [122]. However, the mechanism underlying the association between SEC24A and SERCA in cell death remained unknown.

Difluoromethylornithine (DFMO) is cardioprotective in its pharmacological mechanism [123]. In cardiac hypertrophy rats, DFMO treatment downregulated the Grp75, CypD, and VDAC1 expressions, which indicated MAM signaling pathways. On the contrary, autophagy-associated proteins Atg5 and Beclin1 were upregulated in the heart tissue. These might be the mechanisms of DFMO treatment to achieve a therapeutic effect [124].

MAMs and autophagy have been demonstrated to be negatively correlated in a number of hereditary disorders. The Wolfram syndrome is a rare autosomal recessive disease with functional alterations of MAMs, of which no eradication measures are available at the moment. Lucie discovered that in the wolfram syndrome, activating the Sig-1R might rectify the related rising autophagy and mitophagy as well as counterbalance the contact's stability [125]. Additionally, the nuclear-encoded Glycyl-tRNA synthetase gene (GARS) mutations were described in inheriting neuropathies. In dominant GARS mutations, significant downregulation of the VAPB and its downstream pathway, loosening of the ER-mitochondrial associations, were detected. Nevertheless, larger and more numerous autophagic vacuoles were found in the neuropathy cell line [126]. Mutations in the human cystathionine beta-synthase (CBS) gene reduced MFN2 expression but enhanced receptor-mediated mitophagy and high endothelial cell death [127]. In mammal erythroid differentiation, mitochondria are losing MAM contact sites, and autophagic structures such as autophagosomes increase under electron microscopy [128].

The depletion of TOM70 impaired IP3-linked ER-mitochondrial Ca^2+^ communication, dampened mitochondrial respiration, and resulted in the induction of autophagy [129]. Mitochondrial homeostasis was disrupted by the BAP31-TOM40 complex at MAMs, which triggered AMPK-ULK signaling and induced autophagy [35, 130]. AMPK plays a negative role in MAMs formation through reducing the expression of FUNDC1 and interacts directly with MFN2 to initiate autophagy [131, 132]. Oxidative stress conditions inhibited Sig-1R in MAMs to promote autophagy in cardiomyocytes [133].

The integrity of MAMs and the level of autophagy were found to be trending in the opposite direction in the aforementioned physiological or pathological situations, with the disruption of endoplasmic reticulum and mitochondrial contacts being retained in the same setting as the initiation of autophagy. A generalization of these studies revealed that autophagy was partly actuated by activation of the AMPK pathway. At the same time, a more prevalent cause was shown to be the disruption of calcium ion transport caused by a disorder of the MAMs, stimulating an increase in the level of autophagy.

## 4. Conclusions

A cell is a microscopic world where relatively independent organelles communicate with each other structurally and functionally, and MAMs are tiny nodes in this vast informational network. Despite their tiny size, MAMs are rich in protein composition. They communicate with a wide range of external molecules, providing the basis for their multiple roles in pathological and physiological states. They are intrinsically tied to autophagy.

There is a growing body of evidence indicating MAMs act as a platform for the initiation of autophagosomes. The reduction in autophagy levels caused by disruption of their integrity is associated with the development of a range of diseases. Correction of autophagy through modification of MAMs also has a therapeutic effect on some aging, cardiovascular, and infectious diseases. However, several further investigations have demonstrated that the level of autophagy is negatively linked to the strength of the ER-mitochondria connection. Structural or functional disruptions of MAMs and abnormal autophagy activation have been observed in cancer or multiple toxic agents, as well as in some hereditary diseases. Analysis of the underlying mechanisms concludes that this phenomenon may be tied to the AMPK pathway and altered Ca2+ transport.

MAMs present two faces in autophagy, inseparable from their constituent proteins' complexity and powerful and diverse functions. Various questions remain unresolved regarding the relationship between MAMs and autophagy, such as the specific proteins involved in the expansion of autophagosomes among MAMs that have not been identified, and the exact regulatory mechanism of MAMs on autophagy is still unclear. It is believed that the relationship between MAMs and autophagy will be revealed shortly with the development of related technologies and in-depth research.
